# Crystal structure of fenpropathrin

**DOI:** 10.1107/S160053681402474X

**Published:** 2014-11-19

**Authors:** Gihaeng Kang, Youngeun Jeon, Sangjin Lee, Tae Ho Kim

**Affiliations:** a Department of Chemistry and Research Institute of Natural Sciences, Gyeongsang National University, Jinju 660-701, Republic of Korea

**Keywords:** crystal structure, fenpropathrin, cyclo­propane­carboxyl­ate, pyrethroid insecticide, C—H⋯π inter­actions

## Abstract

In the title compound [systematic name: cyano­(3-phen­oxy­phen­yl)methyl 2,2,3,3-tetra­methyl­cyclo­propane­carboxyl­ate], C_22_H_23_NO_3_, which is the pyrethroid insecticide fenpropathrin, the dihedral angle between the cyclo­propane ring plane and the carboxyl­ate group plane is 88.25 (11)°. The dihedral angle between the benzene and phenyl rings in the phen­oxy­benzyl group is 82.99 (4)°. In the crystal, C—H⋯N hydrogen bonds and weak C—H⋯π inter­actions link adjacent mol­ecules, forming loop chains along the *b*-axis direction.

## Related literature   

For information on the toxicity and insecticidal properties of the title compound, see: Wu *et al.* (1999 ▶); Hall & Nguyen (2010 ▶). For related crystal structures, see: Baert & Guelzim (1991 ▶); Yang *et al.* (2011 ▶).
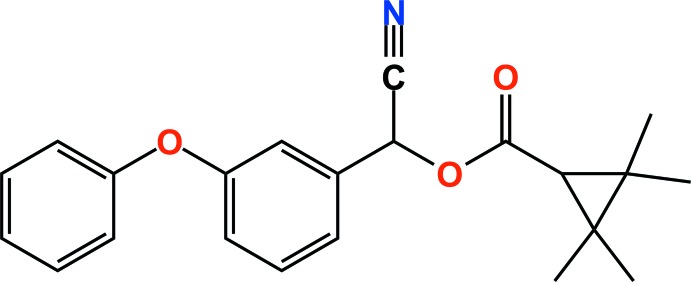


## Experimental   

### Crystal data  

C_22_H_23_NO_3_
*M* *_r_* = 349.41
Monoclinic, 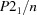

*a* = 16.2578 (4) Å
*b* = 6.4799 (1) Å
*c* = 19.2475 (4) Å
β = 112.453 (1)°
*V* = 1873.99 (7) Å^3^
*Z* = 4
Mo *K*α radiation
μ = 0.08 mm^−1^
*T* = 173 K
0.50 × 0.20 × 0.03 mm

### Data collection  

Bruker APEXII CCD diffractometer
Absorption correction: multi-scan (*SADABS*; Bruker, 2009 ▶) *T*_min_ = 0.960, *T*_max_ = 0.998
30999 measured reflections
4279 independent reflections
3703 reflections with *I* > 2σ(*I*)
*R*_int_ = 0.029

### Refinement  

*R*[*F* ^2^ > 2σ(*F* ^2^)] = 0.039
*wR*(*F* ^2^) = 0.105
*S* = 1.05
4279 reflections
239 parameters
H-atom parameters constrained
Δρ_max_ = 0.28 e Å^−3^
Δρ_min_ = −0.24 e Å^−3^

### 

Data collection: *APEX2* (Bruker, 2009 ▶); cell refinement: *SAINT* (Bruker, 2009 ▶); data reduction: *SAINT*; program(s) used to solve structure: *SHELXTL* (Sheldrick, 2008 ▶); program(s) used to refine structure: *SHELXTL*; molecular graphics: *DIAMOND* (Brandenburg, 2010 ▶); software used to prepare material for publication: *SHELXTL*.

## Supplementary Material

Crystal structure: contains datablock(s) global, I. DOI: 10.1107/S160053681402474X/hg5417sup1.cif

Structure factors: contains datablock(s) I. DOI: 10.1107/S160053681402474X/hg5417Isup2.hkl

Supporting information file. DOI: 10.1107/S160053681402474X/hg5417Isup3.cml

**Fig. 1.**
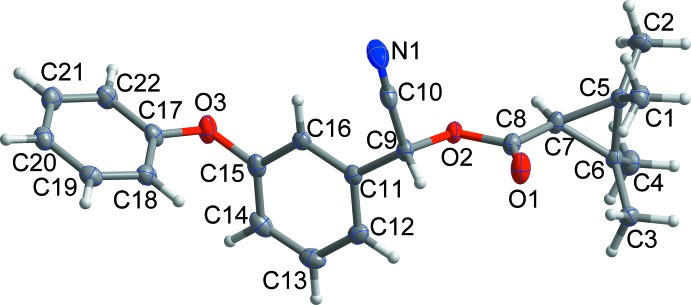
The asymmetric unit of the title compound with the atom numbering scheme. Displacement ellipsoids are drawn at the 50% probability level. H atoms are shown as small spheres of arbitrary radius.

. DOI: 10.1107/S160053681402474X/hg5417fig1.tif
The asymmetric unit of the title compound with the atom numbering scheme. Displacement ellipsoids are drawn at the 50% probability level. H atoms are shown as small spheres of arbitrary radius.

**Fig. 2.**
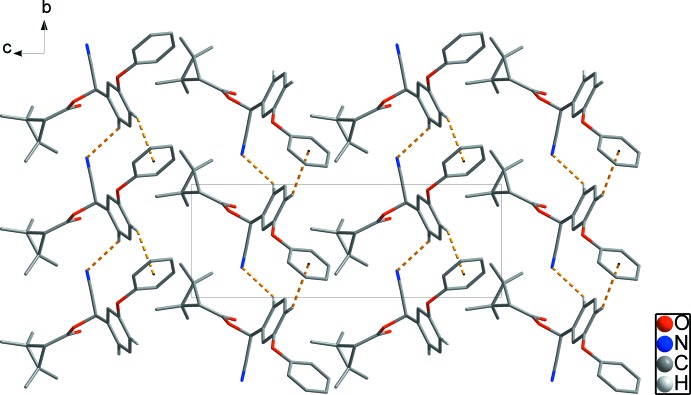
Crystal packing of the title compound with C—H···N hydrogen bonds and weak intermolecular C—H···π interactions are shown as dashed lines. H atoms bonded to C atoms have been omitted for clarity, except H atoms of interactions.

. DOI: 10.1107/S160053681402474X/hg5417fig2.tif
Crystal packing of the title compound with C—H⋯N hydrogen bonds and weak inter­molecular C—H⋯π inter­actions are shown as dashed lines. H atoms bonded to C atoms have been omitted for clarity, except H atoms of inter­actions.

CCDC reference: 1033607

Additional supporting information:  crystallographic information; 3D view; checkCIF report

## Figures and Tables

**Table 1:** Hydrogen-bond geometry (, ) *Cg*1 is the centroid of the C17C22 ring.

*D*H*A*	*D*H	H*A*	*D* *A*	*D*H*A*
C12H12N1^i^	0.95	2.62	3.2715(17)	126
C14H14*Cg*1^i^	0.95	2.87	3.6234(15)	137

Symmetry code: (i) 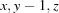.

## supplementary crystallographic information

### S1. Comment

Fenpropathrin, C_22_H_23_NO_3_, is a member of the pyrethroid insecticides and it has been used for controlling insect pests in staple crops such as cereals, potatoes, tobacco, cotton, and fruit (Wu *et al.*, 1999; Hall & Nguyen, 2010). its crystal structure is reported herein. In this compound (Scheme 1, Fig. 1), the dihedral angle between the cyclopropane ring plane and the carboxylate group plane is 88.25 (11)°. The dihedral angle between the benzene and phenyl ring planes in the phenoxybenzyl group is 82.99 (4)°. All bond lengths and bond angles are normal and comparable to those observed in the crystal structure of a similar compound (Baert & Guelzim, 1991; Yang *et al.*, 2011).

In the crystal structure (Fig. 2, Table 1), C12—H12···N1 hydrogen bonds and weak intermolecular C14—H14···*Cg*1 (*Cg*1 is the centroid of the C17–C22 ring) interactions link adjacent molecules, forming loop chains along the *b*-axis direction.

### S2. Experimental

The title compound was purchased from the Dr. Ehrenstorfer GmbH Company. Slow evaporation of a solution in CH_3_OH gave single crystals suitable for X-ray analysis.

### S3. Refinement

All H-atoms were positioned geometrically and refined using a riding model with d(C—H) = 0.98 Å, *U*_iso_ = 1.5*U*_eq_(C) for methyl group, d(C—H) = 0.95 Å, *U*_iso_ = 1.2*U*_eq_(C) for aromatic C—H, and d(C—H) = 1.00 Å, *U*_iso_ = 1.5*U*_eq_(C) for C*sp*^3^—H.

### Crystal data

**Table:**

C_22_H_23_NO_3_	*F*(000) = 744
*M**_r_* = 349.41	*D*_x_ = 1.238 Mg m^−^^3^
Monoclinic, *P*2_1_/*n*	Mo *K*α radiation, λ = 0.71073 Å
Hall symbol: -P 2yn	Cell parameters from 9894 reflections
*a* = 16.2578 (4) Å	θ = 2.7–27.5°
*b* = 6.4799 (1) Å	µ = 0.08 mm^−^^1^
*c* = 19.2475 (4) Å	*T* = 173 K
β = 112.453 (1)°	Block, colourless
*V* = 1873.99 (7) Å^3^	0.50 × 0.20 × 0.03 mm
*Z* = 4	

### Data collection

**Table:**

Bruker APEXII CCD diffractometer	4279 independent reflections
Radiation source: fine-focus sealed tube	3703 reflections with *I* > 2σ(*I*)
Graphite monochromator	*R*_int_ = 0.029
φ and ω scans	θ_max_ = 27.5°, θ_min_ = 2.1°
Absorption correction: multi-scan (*SADABS*; Bruker, 2009)	*h* = −21→21
*T*_min_ = 0.960, *T*_max_ = 0.998	*k* = −8→8
30999 measured reflections	*l* = −24→24

### Refinement

**Table:**

Refinement on *F*^2^	Primary atom site location: structure-invariant direct methods
Least-squares matrix: full	Secondary atom site location: difference Fourier map
*R*[*F*^2^ > 2σ(*F*^2^)] = 0.039	Hydrogen site location: inferred from neighbouring sites
*wR*(*F*^2^) = 0.105	H-atom parameters constrained
*S* = 1.05	*w* = 1/[σ^2^(*F*_o_^2^) + (0.044*P*)^2^ + 0.8901*P*] where *P* = (*F*_o_^2^ + 2*F*_c_^2^)/3
4279 reflections	(Δ/σ)_max_ < 0.001
239 parameters	Δρ_max_ = 0.28 e Å^−^^3^
0 restraints	Δρ_min_ = −0.24 e Å^−^^3^

### Special details

**Table:**

Geometry. All e.s.d.'s (except the e.s.d. in the dihedral angle between two l.s. planes) are estimated using the full covariance matrix. The cell e.s.d.'s are taken into account individually in the estimation of e.s.d.'s in distances, angles and torsion angles; correlations between e.s.d.'s in cell parameters are only used when they are defined by crystal symmetry. An approximate (isotropic) treatment of cell e.s.d.'s is used for estimating e.s.d.'s involving l.s. planes.
Refinement. Refinement of *F*^2^ against ALL reflections. The weighted *R*-factor *wR* and goodness of fit *S* are based on *F*^2^, conventional *R*-factors *R* are based on *F*, with *F* set to zero for negative *F*^2^. The threshold expression of *F*^2^ > σ(*F*^2^) is used only for calculating *R*-factors(gt) *etc*. and is not relevant to the choice of reflections for refinement. *R*-factors based on *F*^2^ are statistically about twice as large as those based on *F*, and *R*- factors based on ALL data will be even larger.

### Fractional atomic coordinates and isotropic or equivalent isotropic displacement parameters (Å^2^)

**Table:**

	*x*	*y*	*z*	*U*_iso_*/*U*_eq_	
O1	0.25941 (6)	0.19890 (17)	0.14087 (5)	0.0316 (2)	
O2	0.11426 (5)	0.23376 (13)	0.12141 (5)	0.01977 (19)	
O3	−0.11802 (6)	0.44803 (17)	0.26198 (5)	0.0300 (2)	
N1	0.13768 (10)	0.73437 (19)	0.16331 (8)	0.0395 (3)	
C1	0.27819 (9)	0.1836 (2)	−0.01169 (8)	0.0265 (3)	
H1A	0.3141	0.1697	0.0421	0.040*	
H1B	0.2674	0.3301	−0.0246	0.040*	
H1C	0.3101	0.1220	−0.0407	0.040*	
C2	0.12837 (9)	0.1063 (2)	−0.11154 (7)	0.0291 (3)	
H2A	0.1523	0.0346	−0.1446	0.044*	
H2B	0.1236	0.2541	−0.1231	0.044*	
H2C	0.0693	0.0510	−0.1195	0.044*	
C3	0.27228 (8)	−0.2137 (2)	0.06589 (7)	0.0239 (3)	
H3A	0.3072	−0.2780	0.0400	0.036*	
H3B	0.2576	−0.3175	0.0964	0.036*	
H3C	0.3071	−0.1027	0.0985	0.036*	
C4	0.12233 (9)	−0.2931 (2)	−0.03358 (8)	0.0271 (3)	
H4A	0.0656	−0.2302	−0.0653	0.041*	
H4B	0.1127	−0.3870	0.0025	0.041*	
H4C	0.1467	−0.3705	−0.0651	0.041*	
C5	0.19010 (8)	0.07394 (19)	−0.03022 (7)	0.0202 (2)	
C6	0.18727 (8)	−0.12554 (19)	0.00844 (6)	0.0193 (2)	
C7	0.14512 (8)	0.06898 (19)	0.02724 (7)	0.0203 (2)	
H7	0.0786	0.0709	0.0040	0.024*	
C8	0.18266 (8)	0.16961 (19)	0.10115 (7)	0.0203 (2)	
C9	0.14081 (8)	0.33957 (19)	0.19286 (7)	0.0196 (2)	
H9	0.2032	0.2990	0.2249	0.024*	
C10	0.13830 (9)	0.5631 (2)	0.17713 (7)	0.0256 (3)	
C11	0.07965 (8)	0.27541 (18)	0.23146 (6)	0.0181 (2)	
C12	0.09429 (8)	0.08420 (19)	0.26710 (7)	0.0222 (3)	
H12	0.1421	−0.0002	0.2672	0.027*	
C13	0.03936 (9)	0.0168 (2)	0.30239 (7)	0.0277 (3)	
H13	0.0498	−0.1136	0.3268	0.033*	
C14	−0.03081 (9)	0.1380 (2)	0.30238 (7)	0.0279 (3)	
H14	−0.0689	0.0914	0.3262	0.034*	
C15	−0.04459 (8)	0.3284 (2)	0.26703 (7)	0.0227 (3)	
C16	0.01002 (8)	0.39989 (19)	0.23177 (6)	0.0193 (2)	
H16	0.0001	0.5315	0.2082	0.023*	
C17	−0.11112 (8)	0.5735 (2)	0.32213 (7)	0.0226 (3)	
C18	−0.03504 (8)	0.5875 (2)	0.38729 (7)	0.0227 (3)	
H18	0.0160	0.5068	0.3932	0.027*	
C19	−0.03484 (9)	0.7219 (2)	0.44377 (7)	0.0245 (3)	
H19	0.0170	0.7332	0.4885	0.029*	
C20	−0.10902 (9)	0.8391 (2)	0.43572 (7)	0.0263 (3)	
H20	−0.1081	0.9307	0.4746	0.032*	
C21	−0.18498 (9)	0.8219 (2)	0.37035 (8)	0.0278 (3)	
H21	−0.2363	0.9013	0.3648	0.033*	
C22	−0.18637 (8)	0.6899 (2)	0.31336 (7)	0.0269 (3)	
H22	−0.2382	0.6789	0.2687	0.032*	

### Atomic displacement parameters (Å^2^)

**Table:**

	*U*^11^	*U*^22^	*U*^33^	*U*^12^	*U*^13^	*U*^23^
O1	0.0183 (4)	0.0463 (6)	0.0292 (5)	−0.0037 (4)	0.0081 (4)	−0.0172 (4)
O2	0.0177 (4)	0.0230 (4)	0.0189 (4)	0.0006 (3)	0.0073 (3)	−0.0055 (3)
O3	0.0168 (4)	0.0493 (6)	0.0232 (5)	0.0028 (4)	0.0069 (4)	−0.0090 (4)
N1	0.0650 (9)	0.0256 (6)	0.0429 (7)	−0.0082 (6)	0.0374 (7)	−0.0051 (5)
C1	0.0277 (7)	0.0259 (7)	0.0297 (7)	−0.0020 (5)	0.0152 (5)	−0.0014 (5)
C2	0.0331 (7)	0.0301 (7)	0.0209 (6)	0.0069 (6)	0.0069 (5)	0.0019 (5)
C3	0.0224 (6)	0.0271 (7)	0.0220 (6)	0.0045 (5)	0.0082 (5)	0.0016 (5)
C4	0.0294 (7)	0.0242 (7)	0.0274 (7)	−0.0033 (5)	0.0104 (5)	−0.0072 (5)
C5	0.0210 (6)	0.0215 (6)	0.0179 (6)	0.0025 (5)	0.0071 (5)	−0.0019 (5)
C6	0.0191 (5)	0.0206 (6)	0.0183 (5)	0.0009 (5)	0.0074 (4)	−0.0030 (5)
C7	0.0159 (5)	0.0233 (6)	0.0210 (6)	0.0009 (4)	0.0064 (4)	−0.0047 (5)
C8	0.0185 (6)	0.0213 (6)	0.0224 (6)	−0.0004 (5)	0.0093 (5)	−0.0031 (5)
C9	0.0195 (6)	0.0208 (6)	0.0189 (6)	−0.0014 (4)	0.0077 (5)	−0.0048 (5)
C10	0.0319 (7)	0.0261 (7)	0.0259 (6)	−0.0057 (5)	0.0190 (5)	−0.0069 (5)
C11	0.0182 (5)	0.0196 (6)	0.0157 (5)	−0.0024 (4)	0.0054 (4)	−0.0035 (4)
C12	0.0241 (6)	0.0191 (6)	0.0203 (6)	0.0001 (5)	0.0050 (5)	−0.0032 (5)
C13	0.0346 (7)	0.0217 (6)	0.0230 (6)	−0.0064 (5)	0.0067 (5)	0.0015 (5)
C14	0.0275 (7)	0.0355 (7)	0.0219 (6)	−0.0117 (6)	0.0107 (5)	−0.0017 (5)
C15	0.0162 (6)	0.0329 (7)	0.0180 (6)	−0.0017 (5)	0.0055 (4)	−0.0057 (5)
C16	0.0195 (6)	0.0213 (6)	0.0161 (5)	0.0001 (5)	0.0056 (4)	−0.0011 (4)
C17	0.0203 (6)	0.0295 (7)	0.0215 (6)	−0.0032 (5)	0.0118 (5)	−0.0003 (5)
C18	0.0193 (6)	0.0266 (6)	0.0233 (6)	−0.0004 (5)	0.0093 (5)	0.0027 (5)
C19	0.0257 (6)	0.0254 (6)	0.0215 (6)	−0.0037 (5)	0.0081 (5)	0.0017 (5)
C20	0.0308 (7)	0.0250 (7)	0.0275 (7)	−0.0035 (5)	0.0162 (5)	−0.0015 (5)
C21	0.0242 (6)	0.0306 (7)	0.0329 (7)	0.0021 (5)	0.0158 (6)	0.0015 (6)
C22	0.0188 (6)	0.0370 (7)	0.0255 (6)	−0.0004 (5)	0.0091 (5)	0.0008 (6)

### Geometric parameters (Å, º)

**Table:**

O1—C8	1.2036 (15)	C7—H7	1.0000
O2—C8	1.3758 (14)	C9—C10	1.4773 (18)
O2—C9	1.4472 (14)	C9—C11	1.5093 (16)
O3—C17	1.3838 (15)	C9—H9	1.0000
O3—C15	1.3953 (15)	C11—C16	1.3919 (16)
N1—C10	1.1402 (18)	C11—C12	1.3919 (17)
C1—C5	1.5143 (17)	C12—C13	1.3833 (18)
C1—H1A	0.9800	C12—H12	0.9500
C1—H1B	0.9800	C13—C14	1.385 (2)
C1—H1C	0.9800	C13—H13	0.9500
C2—C5	1.5170 (17)	C14—C15	1.3852 (19)
C2—H2A	0.9800	C14—H14	0.9500
C2—H2B	0.9800	C15—C16	1.3869 (17)
C2—H2C	0.9800	C16—H16	0.9500
C3—C6	1.5139 (16)	C17—C18	1.3879 (17)
C3—H3A	0.9800	C17—C22	1.3916 (18)
C3—H3B	0.9800	C18—C19	1.3918 (18)
C3—H3C	0.9800	C18—H18	0.9500
C4—C6	1.5150 (17)	C19—C20	1.3831 (19)
C4—H4A	0.9800	C19—H19	0.9500
C4—H4B	0.9800	C20—C21	1.3905 (19)
C4—H4C	0.9800	C20—H20	0.9500
C5—C6	1.5009 (17)	C21—C22	1.3843 (19)
C5—C7	1.5416 (16)	C21—H21	0.9500
C6—C7	1.5423 (16)	C22—H22	0.9500
C7—C8	1.4691 (16)		
			
C8—O2—C9	115.62 (9)	O1—C8—C7	129.17 (11)
C17—O3—C15	118.32 (9)	O2—C8—C7	109.07 (10)
C5—C1—H1A	109.5	O2—C9—C10	107.16 (10)
C5—C1—H1B	109.5	O2—C9—C11	108.90 (9)
H1A—C1—H1B	109.5	C10—C9—C11	113.52 (10)
C5—C1—H1C	109.5	O2—C9—H9	109.1
H1A—C1—H1C	109.5	C10—C9—H9	109.1
H1B—C1—H1C	109.5	C11—C9—H9	109.1
C5—C2—H2A	109.5	N1—C10—C9	177.69 (14)
C5—C2—H2B	109.5	C16—C11—C12	120.00 (11)
H2A—C2—H2B	109.5	C16—C11—C9	122.16 (11)
C5—C2—H2C	109.5	C12—C11—C9	117.84 (11)
H2A—C2—H2C	109.5	C13—C12—C11	120.14 (12)
H2B—C2—H2C	109.5	C13—C12—H12	119.9
C6—C3—H3A	109.5	C11—C12—H12	119.9
C6—C3—H3B	109.5	C12—C13—C14	120.46 (12)
H3A—C3—H3B	109.5	C12—C13—H13	119.8
C6—C3—H3C	109.5	C14—C13—H13	119.8
H3A—C3—H3C	109.5	C13—C14—C15	118.95 (12)
H3B—C3—H3C	109.5	C13—C14—H14	120.5
C6—C4—H4A	109.5	C15—C14—H14	120.5
C6—C4—H4B	109.5	C14—C15—C16	121.61 (12)
H4A—C4—H4B	109.5	C14—C15—O3	120.16 (11)
C6—C4—H4C	109.5	C16—C15—O3	118.11 (12)
H4A—C4—H4C	109.5	C15—C16—C11	118.83 (11)
H4B—C4—H4C	109.5	C15—C16—H16	120.6
C6—C5—C1	119.52 (10)	C11—C16—H16	120.6
C6—C5—C2	119.80 (11)	O3—C17—C18	123.63 (11)
C1—C5—C2	111.82 (11)	O3—C17—C22	115.50 (11)
C6—C5—C7	60.90 (8)	C18—C17—C22	120.87 (12)
C1—C5—C7	120.39 (10)	C17—C18—C19	118.82 (12)
C2—C5—C7	115.78 (10)	C17—C18—H18	120.6
C5—C6—C3	119.66 (10)	C19—C18—H18	120.6
C5—C6—C4	119.97 (10)	C20—C19—C18	120.95 (12)
C3—C6—C4	112.04 (11)	C20—C19—H19	119.5
C5—C6—C7	60.86 (8)	C18—C19—H19	119.5
C3—C6—C7	120.03 (10)	C19—C20—C21	119.50 (12)
C4—C6—C7	115.43 (10)	C19—C20—H20	120.3
C8—C7—C5	123.36 (10)	C21—C20—H20	120.3
C8—C7—C6	122.17 (10)	C22—C21—C20	120.44 (12)
C5—C7—C6	58.24 (7)	C22—C21—H21	119.8
C8—C7—H7	114.0	C20—C21—H21	119.8
C5—C7—H7	114.0	C21—C22—C17	119.42 (12)
C6—C7—H7	114.0	C21—C22—H22	120.3
O1—C8—O2	121.77 (11)	C17—C22—H22	120.3
			
C1—C5—C6—C3	0.51 (16)	O2—C9—C11—C16	−101.40 (12)
C2—C5—C6—C3	145.25 (12)	C10—C9—C11—C16	17.88 (16)
C7—C5—C6—C3	−109.99 (12)	O2—C9—C11—C12	78.15 (13)
C1—C5—C6—C4	−145.25 (11)	C10—C9—C11—C12	−162.58 (11)
C2—C5—C6—C4	−0.52 (17)	C16—C11—C12—C13	0.47 (17)
C7—C5—C6—C4	104.24 (12)	C9—C11—C12—C13	−179.08 (11)
C1—C5—C6—C7	110.50 (12)	C11—C12—C13—C14	0.28 (19)
C2—C5—C6—C7	−104.76 (12)	C12—C13—C14—C15	−0.53 (19)
C6—C5—C7—C8	110.01 (13)	C13—C14—C15—C16	0.04 (19)
C1—C5—C7—C8	0.90 (18)	C13—C14—C15—O3	175.92 (11)
C2—C5—C7—C8	−138.72 (12)	C17—O3—C15—C14	84.56 (15)
C1—C5—C7—C6	−109.10 (12)	C17—O3—C15—C16	−99.42 (13)
C2—C5—C7—C6	111.28 (12)	C14—C15—C16—C11	0.69 (18)
C5—C6—C7—C8	−112.01 (13)	O3—C15—C16—C11	−175.27 (10)
C3—C6—C7—C8	−2.61 (17)	C12—C11—C16—C15	−0.94 (17)
C4—C6—C7—C8	136.39 (12)	C9—C11—C16—C15	178.59 (10)
C3—C6—C7—C5	109.39 (12)	C15—O3—C17—C18	0.13 (18)
C4—C6—C7—C5	−111.60 (12)	C15—O3—C17—C22	179.54 (11)
C9—O2—C8—O1	0.52 (17)	O3—C17—C18—C19	178.79 (12)
C9—O2—C8—C7	−179.00 (10)	C22—C17—C18—C19	−0.60 (19)
C5—C7—C8—O1	−30.9 (2)	C17—C18—C19—C20	0.34 (19)
C6—C7—C8—O1	39.8 (2)	C18—C19—C20—C21	0.21 (19)
C5—C7—C8—O2	148.58 (11)	C19—C20—C21—C22	−0.5 (2)
C6—C7—C8—O2	−140.71 (11)	C20—C21—C22—C17	0.3 (2)
C8—O2—C9—C10	94.85 (12)	O3—C17—C22—C21	−179.15 (12)
C8—O2—C9—C11	−141.98 (10)	C18—C17—C22—C21	0.3 (2)

### Hydrogen-bond geometry (Å, º)

*Cg*1 is the centroid of the C17–C22 ring.

**Table:**

*D*—H···*A*	*D*—H	H···*A*	*D*···*A*	*D*—H···*A*
C12—H12···N1^i^	0.95	2.62	3.2715 (17)	126
C14—H14···*Cg*1^i^	0.95	2.87	3.6234 (15)	137

Symmetry code: (i) *x*, *y*−1, *z*.

## References

[bb1] Baert, F. & Guelzim, A. (1991). *Acta Cryst.* C**47**, 606–608.

[bb2] Brandenburg, K. (2010). *DIAMOND*. Crystal Impact GbR, Bonn, Germany.

[bb3] Bruker (2009). *APEX2*, *SAINT* and *SADABS*. Bruker AXS Inc., Madison, Wisconsin, USA.

[bb4] Hall, D. G. & Nguyen, R. (2010). *BioControl*, **55**, 601–611.

[bb5] Sheldrick, G. M. (2008). *Acta Cryst.* A**64**, 112–122. 10.1107/S0108767307043930 18156677

[bb6] Wu, W. Z., Xu, Y., Schramm, K.-W. & Kettrup, A. (1999). *Ecotox. Environ. Safe.* **42**, 203–206. 10.1006/eesa.1998.1743 10090808

[bb7] Yang, H., Kim, T. H., Park, K.-M. & Kim, J. (2011). *Acta Cryst.* E**67**, o1275. 10.1107/S1600536811014760 PMC3089073 21754558

