# Supplementary figures and images for: Crystal structure of fenpropathrin
Source: Acta Crystallogr Sect E Struct Rep Online. 2014 Nov 19;70(Pt 12):o1265. doi: 10.1107/S160053681402474X (PMC4257423; doi:10.1107/S160053681402474X)

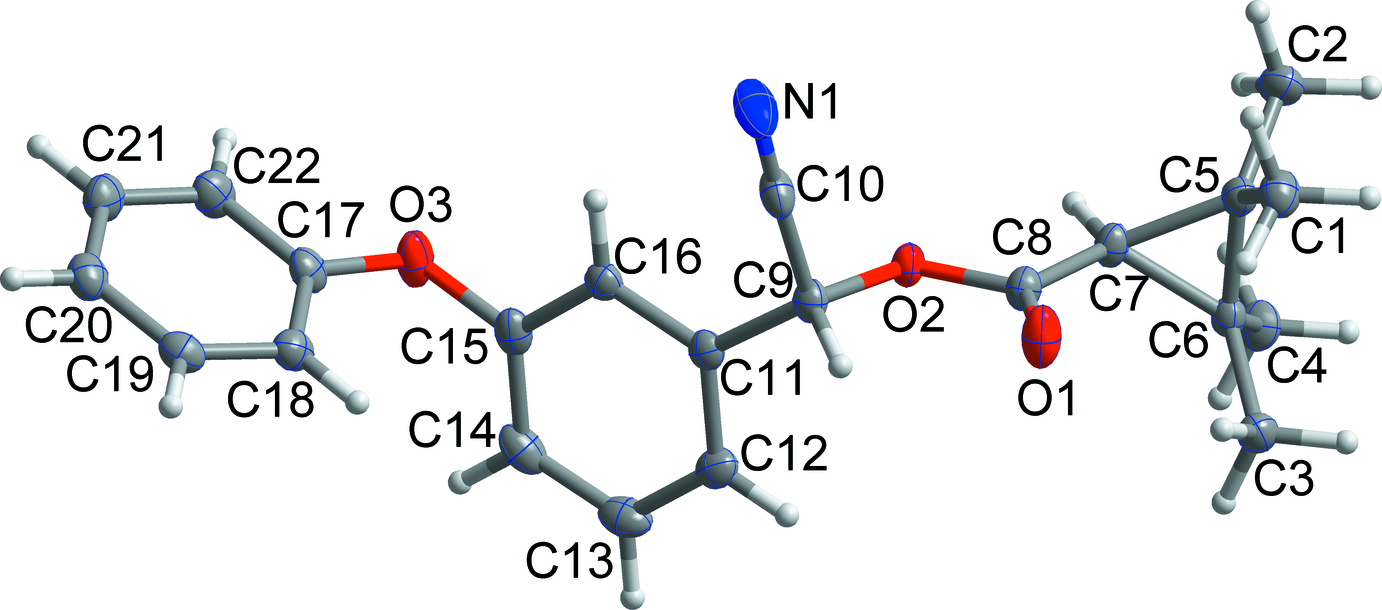

Supplement: Supplementary file 4 [file e-70-o1265-fig1.tif]

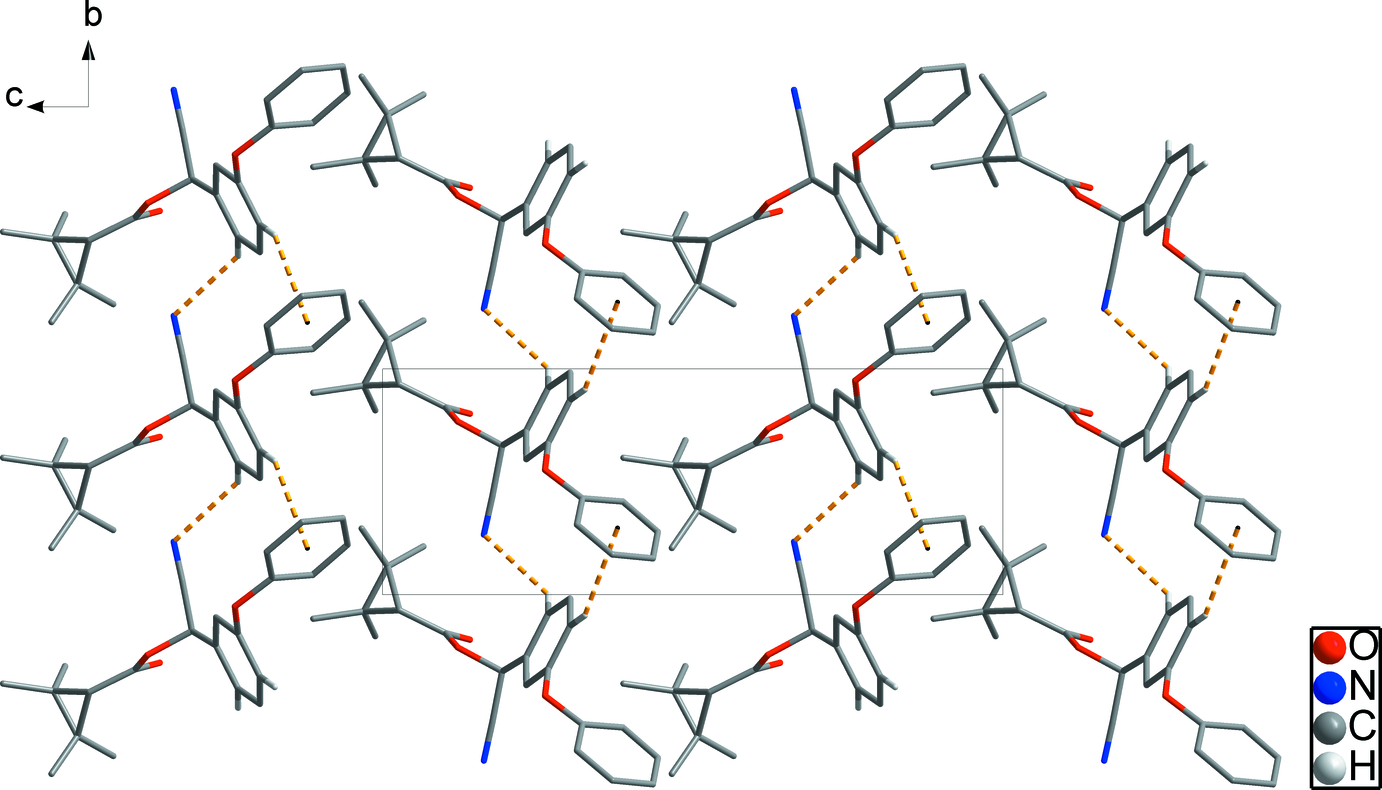

Supplement: Supplementary file 5 [file e-70-o1265-fig2.tif]
